# ASSESSING AGE INCLUSIVITY USING THE AGE-FRIENDLY INVENTORY AND CAMPUS CLIMATE SURVEY

**DOI:** 10.1093/geroni/igae098.1586

**Published:** 2024-12-31

**Authors:** Jacqueline Eaton, Katarina Friberg-Felsted, Susan Whitbourne

**Affiliations:** Chair; Co-Chair; Discussant

## Abstract

During a time of increased focus on diversity, equity, and inclusion in institutions of higher education, age inclusion remains largely unexplored. The Age-Friendly Inventory and Campus Climate Survey (ICCS) is a tool that institutions of higher education can use to identify strengths and weaknesses specific to age-friendliness. This instrument measures age inclusivity by comparing age-friendly practices with student, staff, faculty, and lifelong learners’ perceptions of these practices. This symposium explores the process of using the ICCS at four different universities to understand the methods used to target each institution’s unique needs, with the goal of improving age-friendliness in higher education: 1) Zimmer et al., present the outcomes of using the ICCS to establish a baseline of age-friendliness at the University of Calgary and the process to generate action items from the results to develop a plan to enhance age-friendliness. 2) Waterhouse et al., describes the process of customizing the ICCS to meet the unique needs of an academic medical campus at the University of Colorado Anschutz Medical Campus. 3) Zisberg et al., report on the experience of translating the ICCS into Hebrew and the subsequent results implementing the tool at the University of Haifa. 4) Eaton & Friberg-Felsted explore the use of the ICCS within the College of Nursing at the University of Utah to identify ways to meet the learning needs of age diverse groups. Dr. Susan Whitbourne, from the team that developed the ICCS, will facilitate discussion surrounding age inclusivity in higher education.

